# Preparedness for further boosting coronavirus disease 2019 vaccines

**DOI:** 10.1002/ctd2.55

**Published:** 2022-04-29

**Authors:** So Yun Lim, Jiwon Jung, Sung‐Han Kim

**Affiliations:** ^1^ Department of Infectious Diseases Asan Medical Center University of Ulsan College of Medicine Seoul South Korea; ^2^ Office for Infection Control Asan Medical Center Seoul South Korea

**Keywords:** COVID‐19, SARS‐CoV‐2, vaccine

## 1 COMMENTARY

More than a year has elapsed since coronavirus disease 2019 (COVID‐19) vaccines were rolled out. Early data have revealed that COVID‐19 mRNA vaccine efficacy or effectiveness against symptomatic COVID‐19 was approximately 95% and that against the asymptomatic form was approximately 90%.^1^ However, these data are limited owing to a short (3–4 months) follow‐up period after vaccination before the emergence of highly transmissible or immune evasive variants, such as the delta or omicron variants. After the emergence of the delta variant, we observed an unexpectedly high rate of breakthrough infection, even in fully vaccinated individuals; this was inconsistent with the observed rate of breakthrough infections of measles or varicella, considering their vaccines have similar effectiveness. The fundamental difference between COVID‐19 and measles or varicella is the causative virus incubation period, which is 5 days in COVID‐19^2^ versus 10–14 days in measles or varicella.^3^

Circulating neutralizing antibody levels from long‐lived plasma cells in the bone marrow would inevitably decrease several months after vaccination; however, memory B cells, which do not produce any antibody themselves, may maintain and readily proliferate and differentiate into high‐affinity plasma cells within 4–7 days after re‐exposure.^4^ Thus, the incubation or latent period of severe acute respiratory syndrome coronavirus 2 (SARS‐CoV‐2) is too short for SARS‐CoV‐2‐specific memory B cells to overcome the infection itself, although this rapid memory response might prevent progression to severe COVID‐19. Therefore, SARS‐CoV‐2 infection may largely depend on the circulating neutralizing antibody levels, so the waning antibody titers would be inevitably correlated with the susceptibility of breakthrough infection after COVID‐19 vaccination. So, the decrease in symptomatic COVID‐19 protection has been well linked to a waning antibody response following a two‐dose COVID‐19 vaccination regimen.^5^ Further, the emergence of SARS‐CoV‐2 variants that can evade vaccine‐induced immunity facilitated the breakthrough infection, since circulating neutralizing antibody levels against these immune‐evasive variants were lower.^6^ For these reasons, breakthrough infection rates sharply increased 6 months after the two‐dose COVID‐19 vaccination drive in the delta‐variant dominant era.^6^

Neutralizing antibody levels against the delta variant after two‐dose COVID‐19 vaccination declined more rapidly than those after natural SARS‐CoV‐2 infection.^6^ Thus, the breadth and durability of neutralizing antibodies might be wider and longer, respectively, after natural SARS‐CoV‐2 infection than after COVID‐19 vaccination. However, the new, highly transmissible omicron variant can easily evade natural infection‐induced immunity.^7^ Furthermore, the two‐dose COVID‐19 vaccination was suboptimal to overcoming the omicron infection^7^; thus, many countries experienced huge omicron waves.

To overcome the breakthrough infection, more durable and wider breadth of neutralizing antibodies are needed. One strategy may be ensuring a longer interval of COVID‐19 vaccine boosting because longer intervals are associated with generally greater immune responses.^4^ However, further studies are needed on whether waning immunity and antibody breadth after a booster dose of COVID‐19 vaccine with longer intervals is more favourable than that after two doses of COVID‐19 vaccine with short intervals. Another strategy may be administering booster doses of COVID‐19 vaccines manufactured from the different variants. However, the breadth and durability of these heterologous antigenic booster doses are largely unknown. Last, COVID‐19 vaccination followed by SARS‐CoV‐2 infection or vice versa would induce a more beneficial immune response than only vaccine‐induced immune response.^8^ Further studies are urgently needed on whether an individualized booster dose administration schedule is needed depending on the patient's SARS‐CoV‐2 infection.

In conclusion, new variants that are highly transmissible and demonstrate great immune‐evasive ability may emerge in the upcoming months, and waning neutralizing antibody levels with decreasing herd immunity is expected. Thus, there is a need to urgently design a new boosting strategy, in terms of timing and vaccine types with/without consideration of the prior history of SARS‐CoV‐2 infection.
